# Enhancing grape disease detection: A comparative analysis of hybrid CNN-LSTM and CNN methods

**DOI:** 10.1016/j.mex.2026.103983

**Published:** 2026-06-02

**Authors:** Vinod Mulik, Vinod Patil

**Affiliations:** a Bharati Vidyapeeth’s (Deemed to be University) College of Engineering, Pune, 411043, India; b Bharati Vidyapeeth’s College of Engineering for Women, Pune, 411043, India

**Keywords:** Agricultural productivity, Hybrid method, Balanced dataset, Imbalanced dataset, Computational cost

## Abstract

Crop disease detection is crucial for maintaining high agricultural productivity and minimizing the financial losses of farmers.

• This study compares the performance of a hybrid method based on integrating the Long Short-Term Memory algorithm (LSTM) and Convolutional Neural Network (CNN) with a standalone CNN method for grape crop disease detection and classification.
• The proposed methods are trained and tested on a comprehensive balanced and imbalanced grape leaf dataset with 4000 and 4062 images, respectively. The imbalanced dataset has 423 images of the healthy class, 1383 of ESCA, 1076 of leaf blight, and 1180 images of the black rot class, while the balanced dataset has 1000 images in each class
• The LSTM-CNN method achieved 100%, and the CNN method achieved 99.47% accuracy on the balanced dataset. Also, in the case of the imbalanced dataset, 100% and 97.89%, respectively.

Among these two methods implemented, the proposed LSTM-CNN method has achieved excellent results in terms of performance metrics. This study presents a deep learning-based system for automated leaf disease classification. The model is designed for end-user deployment, where users can upload a leaf image and receive an immediate prediction of the disease without requiring model retraining or technical expertise.

## Graphical abstract

**Figure:**
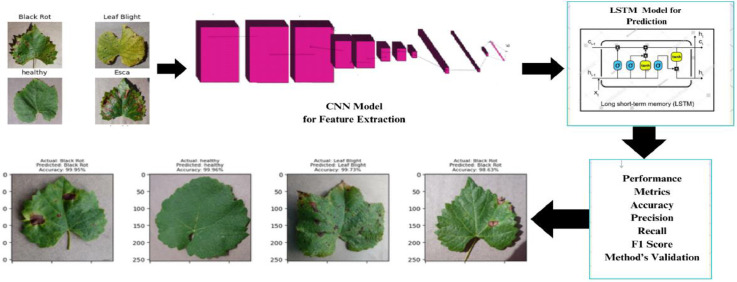

## Nomenclature

Abbreviation: Description
LSTM-CNN: Long Term Short Memory – Convolutional Neural Network
CNN: Convolutional Neural Network
CARAFE: Content-aware Reassembly of Features
AKConv: Adaptive Kernel Convolution
YOLOv8: You Only Look Once version 8
IoU: Intersection over Union
ResNet: Residual Neural Network
CBAM: Convolutional Block Attention Module
GAN: Generative Adversarial Network
SVM: Support Vector Machine
CL: Convolution Layer
MP: Max Pooling Layer
DL: Dense Layer
FL: Flatten Layer

## Specifications table

**Table:** 

**Subject area**	Agricultural and Biological Sciences
**More specific subject area**	*Crop disease detection and classification using artificial intelligence methods.*
**Name of your method**	Integration of LSTM with CNN and a standalone Convolutional Neural Network (CNN)
**Name and reference of the original method**	P. Gaur, R. Sharma, R. Kumar, A. Gupta, R. Sharma and V. Kukreja, "Fighting Grape Black Rot with Deep Learning: A CNN-LSTM Hybrid Method for Disease Severity Classification," 2023 4th International Conference for Emerging Technology (INCET), Belgaum, India, 2023, pp. 1–5, doi: 10.1109/INCET57972.2023.10170159.
**Resource availability**	Plant Village Image Dataset Grape Leaf Dataset – Balanced (https://www.kaggle.com/datasets/tejaswim04/grape-dataset-equal) Grape Leaf Dataset – Imbalanced (https://www.kaggle.com/datasets/tejaswim04/grape-disease-dataset)

## Background

A popular crop in the horticultural sector, grapes are highly valued for their distinct flavor and health advantages. Nevertheless, this crop is extremely vulnerable to many illnesses that can drastically lower output and quality. Farmers face major challenges when plant diseases and pests go unnoticed. These threats can reduce crop yields, leading to significant financial losses. If not detected early, they can destroy over 45% of crops yearly, even when pesticides are used [1]*.* The hybrid method was used in grape leaf disease detection using the LSTM and CNN for the black rot class. This method used 10,000 images of the black rot class and achieved 93.06% accuracy [2]. The hybrid method combining CNNs and vision transformers was employed to classify grape leaves and diagnose grape diseases from digital images. The study utilizes the Plantvillage and Grapevine datasets and evaluates 14 CNN and 17 vision transformer methods, achieving up to 100% accuracy in specific classes [3]. An improved YOLOv8-based method was employed for detecting grape leaf diseases. This study focused on introducing the AKConv module for flexible convolution, the Coordinate Attention (CA) mechanism, the CARAFE upsampling module, and the Wise-IoU loss function to improve the performance in detection. This method has reduced computation cost by 2.8 M parameters, obtaining an F1 score of 92.4% [4]. Another hybrid method was applied for grape leaf disease detection using Inception-ResNet with CBAM for local feature extraction, Shuffle-Transformer for global feature extraction, and Coordinate Attention for the classification fusion technique. The method achieved 99.56% accuracy [5]. In [6], an improved Convolutional Neural Network (CNN) method was implemented for classifying grape leaf diseases. The Enhanced Convolution method integrates depthwise separable convolution and inverted residual blocks to enhance computational efficiency and accuracy. The [7] proposed a Fine Grained-GAN, a generative adversarial network (GAN)-based method that boosts the dataset by generating synthetic leaf spot images. This method is useful if limited samples are available for training. The Res-Net 50 algorithm used for classification achieves 96.27% accuracy. Another hybrid method was proposed in [8] using K-means clustering and a multiclass SVM algorithm for feature extraction and classification, respectively. The principal component analysis was applied to reduce computational dimensions. The method achieved 98.97% accuracy. An integrated method was applied in grape disease detection using multimodal input data (images from the dataset and sensor data) and parallel heterogeneous activation functions. This method enhances the robustness and accuracy of detection. This method achieved 91% accuracy [9]. The hybrid method integrated K-means clustering, and the SVM algorithm was used to detect powdery mildew and downy mildew in grapes. In this method, K-means clustering was used for segmentation and feature extraction, and SVM was used for classification. This method achieved 88.89% accuracy [10].

## Method details

### Experimental set-up

The training and testing of these models were done on the Kaggle platform. These two models were implemented on the Kaggle platform using Tensor Flow 2.x and Keras APIs. Each model is compiled using the Adam optimizer with a learning rate of 0.0001 and trained over 50 epochs with 32 batch size. Data pre-processing, augmentation, and performance evaluation (including accuracy, precision, recall, and F1-score) were also conducted within the Kaggle environment, employing GPU-based acceleration for rapid training.

### Data collection

The dataset is downloaded from the PlantVillage dataset, which includes healthy and diseased leaves. The grape dataset has four classes: healthy, ESCA, Leaf Blight, and Black Rot. (Table 1) summarizes the dataset distribution for the proposed methods. The first column represents the dataset type and remaining columns with their dataset size for each class. This dataset has 4000 images in the balanced dataset and 4062 in the imbalanced dataset. The balanced nature of this dataset ensures that each disease class is well-represented by 1000 images from each class, allowing the method to learn patterns from all disease classes without bias. On the other hand, the imbalanced dataset contains 4062 images with an unequal distribution across different disease categories. This dataset consists of 423 images of the healthy class, 1383 images of ESCA, 1076 images of leaf blight, and 1180 images from the black rot class. Each image in both datasets is of 256×256 in size.

**Table 1:** Summary of the grape leaf disease dataset.

Dataset Type	No. of Images	Healthy Class	ESCA Class	Leaf Blight Class	Black Rot Class
Balanced Dataset	4000	1000	1000	1000	1000
Imbalanced Dataset	4062	423	1383	1076	1180

### Training, validation, and testing dataset

**Fig. 1:**
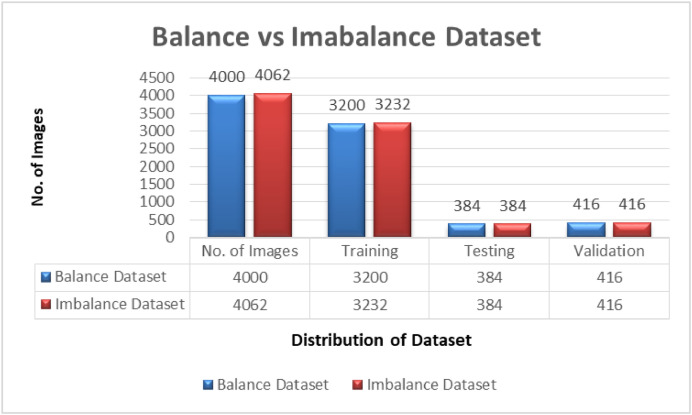
Distribution of the dataset for training, validation, and testing.

This research uses balanced and imbalanced datasets for training, testing, and validation. Fig. 1 illustrates the graphical representation of the dataset distribution across all classes for both balanced and imbalanced datasets. The balanced dataset consists of 4000 images indicated by the blue bar in Fig. 1, evenly distributed across four classes. Of these, 80% of images are designated for training the method, and the remaining samples are split equally, with 10% allocated for testing and another 10% for validation. Like a balanced class, the same distribution was applied for training, testing, and validation for an imbalanced dataset as represented by a red bar in Fig. 1. The uneven class distribution in this dataset may lead to biased predictions, favoring classes with more samples, which could impact the method’s performance and accuracy. In both scenarios, training data is crucial for method learning, while testing and validation data are necessary to evaluate the method's generalization and fine-tune its hyperparameters.

Overall, the dataset effectively conveys the complexity and diversity of classification within grape crops. This substantial dataset indicates a robust foundation for machine learning applications, enabling the development of accurate classification methods. Offering a variety of instances for the method to learn with multiple images can enhance classification accuracy and the method's ability to generalize.

### The architecture of the LSTM-CNN hybrid method

**Fig. 2:**
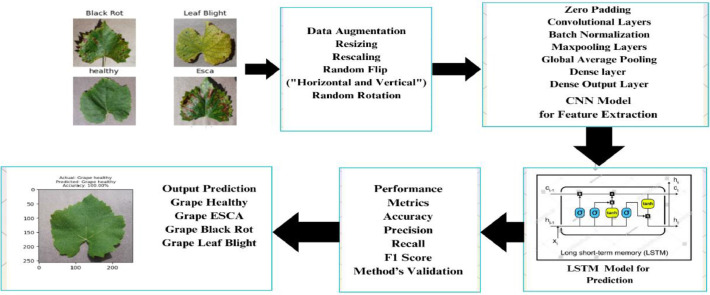
Architecture of LSTM-CNN hybrid method.

This section effectively discusses the architecture of the LSTM-CNN Hybrid method for grape crop disease detection and classification, as shown in Fig. 2.

#### LSTM-CNN hybrid method

The Hybrid LSTM-CNN method starts with a ZeroPadding2D layer, essential for maintaining spatial dimensions before convolution operations. The initial convolutional layer, featuring 32 filters with a 3 × 3 kernel, focuses on extracting low-level features like edges and textures, followed by Batch Normalization to ensure stable training and ReLU activation to introduce non-linearity. A Maxpooling layer with a 2 × 2 kernel size reduces the spatial dimensions while preserving crucial information. A second convolutional layer with 128 filters and a 3 × 3 kernel delves deeper into pattern extraction, utilizing Batch Normalization, ReLU activation, and Maxpooling. A third convolutional layer, with 180 filters and a 3 × 3 kernel, captures even more abstract and high-level features, adhering to the same processing steps.

Once feature extraction is complete, a Global Average Pooling (GAP) layer condenses the spatial dimensions into a single vector for each feature map, effectively summarizing the extracted features. The flatten layer and global average pooling layer were used for 50 epochs, but GAP gave the best results. This approach minimizes the number of parameters and prevents overfitting. The CNN architecture is a feature extractor, setting the stage for sequential modeling with LSTM layers. The LSTM method starts with a Lambda layer that adjusts the input dimensions to make them compatible with the LSTM layer. The LSTM layer features 128 units and uses a tanh activation function, which allows it to learn intricate temporal dependencies from sequential data while keeping gradients stable. With return sequences set to False, the LSTM produces only the final hidden state instead of a full sequence. To combat overfitting, a Dropout layer with a rate of 0.2 is included, randomly setting 20% of the units to zero during training to enhance generalization.

#### CNN architecture

**Fig. 3:**
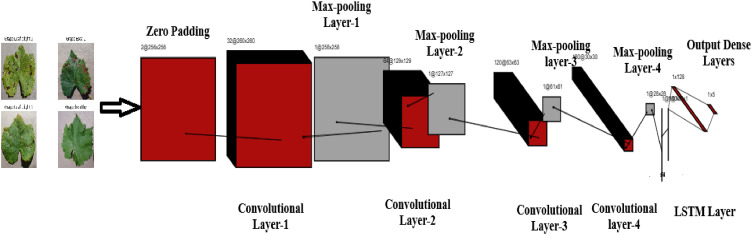
Architecture of proposed CNN method.

A 12 Layer Convolutional Neural Network (CNN) method for Multiclass Crop Disease Detection was designed to efficiently detect and classify a variety of diseases that impact different crops as shown in Fig. 3. This method makes use of a CNN architecture with 12 layers, which usually consists of several convolutional layers with activation functions, pooling layers, and finally fully connected layers. Convolutional layers are necessary to automatically extract features from input photos of crops and analyze patterns in pixel intensities that correspond to illness indicators; While pooling layers assist in downsampling the feature maps, lowering computational load, and enabling the method to collect crucial qualities while preserving pertinent information, each convolutional layer is outfitted with filters that learn to recognize particular attributes. This CNN method's design notably aims to manage multiclass classification, differentiating between healthy and diseased leaves that could exhibit comparable visual symptoms. This architecture uses 1 zero padding layer, 4 convolutional layers, 4 max-pooling layers, 1 flatten layer, and 2 dense layers. The activation function used in this architecture is RELU. One of the most common options for hidden layers in neural networks is the ReLU (Rectified Linear Unit) function acting as an activation function. It introduces non-linearity by directly outputting the input value if it is positive and zero if it is negative. This effectively helps to overcome the vanishing gradient problem and enables faster learning in deep learning methods.

The primary objective of this study is to develop a model that can be directly used by end-users for automated plant disease identification. Accordingly, the model is trained once using a curated dataset and is then deployed in a static form for inference. End-users are expected to provide only a leaf image, and the system returns the predicted disease class. No retraining or fine-tuning is required at the user level. To support this, we provide an inference pipeline that processes a new image and feeds it through the trained model for prediction.

## Method validation

### Mathematical modeling

All the images in the dataset are 256* 256 pixels in size. Now, let us see the layer-wise mathematical detailing of each proposed method shown in Table 2, Table 3. The suggested LSTM-CNN hybrid architecture is described layer by layer in Table 2. A particular layer in the model is described in each row, along with the parameters and operation type that go with it. While the 'Operation' column describes the function carried out (e.g., convolution, pooling, LSTM, etc.), the 'Layer' column specifies the order of layers used. The dimensionality of the data entering and leaving the layer is indicated by "Input Size" and "Output Size," which are commonly expressed as (time steps × features) for LSTM layers or (height × width × channels) for CNN layers. "Kernel Size" determines the spatial size of the filters and is applicable to pooling and convolutional layers. In order to preserve output dimensions, "Padding" indicates whether and how input data is padded (e.g., "same" or "valid"). This thorough analysis shows how the data changes and moves through the hybrid network.

**Table 2:** Layer-wise detailing of the proposed LSTM-CNN hybrid method.

Layer	Operation	Input Size	Kernel Size	Padding	Output Size
Zero Padding Layer	32 filters	256×256	-	2	260×260
CL-1	3 × 3 kernel, 32 filters,	260×260	3 × 3	0	258×258
MP- 1	2 × 2 kernel	258×258	2 × 2	0	129* 129
CL-2	3 × 3 kernel, 64 filters,	129* 129	3 × 3	0	127×127
MP- 2	2 × 2 kernel	127×127	2 × 2	0	63 * 63
CL-3	3 × 3 kernel, 120 filters,	63 * 63	3 × 3	0	61×61
MP- 3	2 × 2 kernel	61×61	2 × 2	0	30×30
CL-4	3 × 3 kernel, 180 filters,	30×30	3 × 3	0	28×28
MP- 4	2 × 2 kernel	28×28	2 × 2	0	14×14
Global Averaging Layer	Converts 3D to 1D	180	-	-	180
Lambda Layer		180	180	-	180
LSTM		180	128	-	158,208
DL- 1	Fully Connected Layer	128	128	-	16,512
DL- 2	Fully Connected Layer	128	3	-	387

**Table 3:** Layer-wise detailing of the proposed CNN method.

Layer	Operation	Input Size	Kernel Size	Stride	Padding	Output Size
Zero Padding Layer	32 filters	256×256	-	-	2	260×260
CL-1	4 × 4 kernel, 32 filters, stride=1	260×260	4 × 4	1	0	256×256
MP- 1	2 × 2 kernel, stride=2	256×256	2 × 2	2	0	128* 128
CL-2	4 × 4 kernel, 64 filters, stride=1	128×128	4 × 4	1	0	125×125
MP- 2	2 × 2 kernel, stride=2	125×125	2 × 2	2	0	62 * 62
CL-3	4 × 4 kernel, 120 filters, stride=1	62 * 62	4 × 4	1	0	59×59
MP- 3	2 × 2 kernel, stride=2	59×59	2 × 2	2	0	29×29
CL-4	4 × 4 kernel, 180 filters, stride=1	29×29	4 × 4	1	0	26×26
MP- 4	2 × 2 kernel, stride=2	26×26	2 × 2	2	0	13×13
FL	Converts 3D to 1D	13×13×180	-	-	-	30,420
DL- 1	Fully Connected Layer	30,420	100	-	-	100
DL- 2	Fully Connected Layer	100	5 (Depends on classes)	-	-	5

The hybrid method architecture shown in Table 2 includes several convolutional and pooling layers, integrating in a hybrid LSTM-CNN structure designed for sequential feature learning. It starts with a zero-padding layer that increases the input dimensions from 256 × 256 to 260 × 260. The initial convolutional layer utilizes 32 filters of size 3 × 3, resulting in a feature map of 258 × 258, which is then reduced to 129 × 129 through max pooling (2 × 2). The second convolutional layer, equipped with 64 filters, yields a 127 × 127 output, further down-sampled to 63 × 63 via max pooling. The third convolutional layer, featuring 120 filters, produces a 61 × 61 output, which is subsequently pooled to 30 × 30. The fourth convolutional layer, using 180 filters, generates a 28 × 28 output, which is max-pooled to 14 × 14. A global averaging layer condenses the feature map into a 1D vector of 180 dimensions, which is processed through a Lambda layer before being input into an LSTM with 128 units, resulting in an output of 158,208. The first dense layer, containing 128 neurons, converts the LSTM output into a 128-dimensional feature space (16,512 parameters), followed by a final dense layer that translates these features into 3 output classes (387 parameters) for classification.

Table 3 shows the deep learning method based on a Convolutional Neural Network (CNN) architecture, which includes several convolutional and pooling layers that lead into fully connected layers for classification. The suggested CNN architecture is thoroughly summarized in this table. In addition to important parameters like input size, kernel size, stride, padding, and output size, it contains the name and operation of each layer. The format (height × width × channels) describes the dimensionality of the feature maps and shows the input and output sizes. Whereas stride describes the step size at which the filter traverses the input, kernel size describes the spatial dimensions of the filters used in convolutional or pooling layers. Whether zero-padding is used to preserve the spatial dimensions is specified by padding. This structure aids in comprehending how data is transformed at each layer of the network. It starts with a zero-padding layer that increases the input dimensions from 256 × 256 to 260 × 260. The first convolutional layer employs a 4 × 4 kernel with 32 filters and operates with a stride of 1, resulting in a 256 × 256 feature map. A 2 × 2 max pooling layer with a stride of 2 reduces this to 128 × 128. The second convolutional layer, featuring 64 filters and a 4 × 4 kernel, produces a 125 × 125 feature map, which is subsequently down-sampled to 62 × 62 through max pooling. The third convolutional layer, equipped with 120 filters, generates a 59 × 59 feature map, which is then pooled down to 29 × 29. The fourth convolutional layer, utilizing 180 filters, yields a 26 × 26 feature map, and the final pooling operation downsamples it to 13 × 13. A flattening layer transforms the 13 × 13 × 180 output into a 1D vector containing 30,420 features. This vector is then processed through a fully connected (dense) layer with 100 neurons, culminating in a dense output layer with 5 neurons that predicts the class labels.

### Hyperparameters

Hyperparameters play a vital role in machine learning, greatly affecting how well a method performs and how efficiently it operates. Table 4 gives the hyperparameters used in the proposed methods, with parameters in the first column and their values in the second column. The way a method learns from data is determined by its hyperparameters. For example, the speed and quality of learning in neural networks are determined by hyperparameters like learning rate, batch size, and number of layers. To get the best method performance, these hyperparameters must be properly adjusted. The batch size used for method training is 32. The image size is 256×256 pixels. The learning rate was set to 0.0001, and a total of 50 epochs was used during training for both datasets. Both datasets were split into three parts: training, testing, and validation, with an 80:10:10 ratio.

**Table 4:** Hyperparameters.

Batch Size	32 Images
Image Size	256×256 Pixels
Channels	3 (RGB)
Learning Rate	0.0001
No. of Epochs	50
Training Dataset Size	80%
Testing Dataset Size	10%
Validation Dataset Size	10%

### Performance analysis

Let us see the details of trainable parameters for the proposed methods.

#### Zero padding layer

Input shape: H × W

Padding: p = 2

(1) Outputsize=(H+2p)×(W+2p)

Notations:

H – Height of Image Input

W– Width of Image Input

P = Padding

#### Convolutional layer equation

The output spatial dimensions for each convolutional layer are given by:

(2) $$O_{W}=\left(\left(I_{W}-K_{W}+2P\right)/S\right)+1$$

(3) $$O_{H}=\left(\left(I_{H}-K_{H}+2P\right)/S\right)+1$$

(4) $$\text{Trainable}\text{Parameters}=\left(K_{W}\times K_{H}\times C_{\text{in}\;}+1\right)\times C_{\text{out}\;}$$

Notations:

O_W_, O_H_ = Output width and height

I_W_, I_H_ = Input width and height

K_W_, K_H_ = Kernel width and height

P = Padding

S = Stride

Cin = Number of input channels

Cout = Number of output filters

Bias term (+1) is included for each output filter

#### LSTM layer equation

The number of trainable parameters in an LSTM layer is given by:

(5) P=4×[((F+L)×L)+L]

Notations:

P = Total trainable parameters

F = Number of input features (input size to LSTM)

L = Number of LSTM units (128 in this case)

The factor 4 accounts for the four LSTM gate computations:

Input gate (i)

Forget gate (f)

Cell state (c)

Output gate (o)

#### Dense layer equation

For a fully connected (dense) layer, the number of trainable parameters is given by:

(6) P=(N×M)+M

Notations:

P = Total trainable parameters

N = Number of input neurons

M = Number of output neurons

Bias term (+M) is included for each output neuron

Table 5 presents trainable and non-trainable parameters across the proposed methods: LSTM-CNN and CNN represented in the first column. Second and third column illustrates the trainable and non-trainable parameters. Among these, the CNN method stands out with the highest count of trainable parameters at 3546,896, and it has no non-trainable parameters, which means it is entirely trainable. In contrast, the LSTM-CNN method features 476,703 trainable parameters along with 808 non-trainable parameters, indicating the presence of an LSTM layer that adds a bit of complexity; however, the hybrid method has reduced the size of trainable parameters and computational cost.

**Table 5:** Method-wise parameters trained.

Name of the Method	Trainable Parameters	Non-trainable Parameters
LSTM-CNN	476,832	808
CNN	3546,896	0

## Results and discussion

**Fig. 4:**
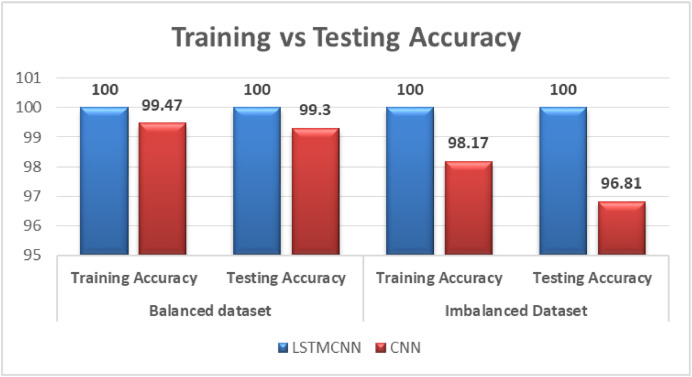
Training accuracy vs testing accuracy.

This section presents the details of the results obtained for the proposed methods. The proposed methods are analyzed using the following performance parameters: Accuracy, Precision, Recall, and F1-Score. A thorough performance analysis of classification methods, including key metrics that gauge how well they classify diverse crops, is presented in Tables 6–8.​ Table 6 and Fig. 4 illustrate the comparison of training and testing accuracy of both methods for balanced and imbalanced datasets. These metrics demonstrate the effectiveness of the proposed methods in accurately detecting and classifying diseases. Table 6 and Fig. 4 show the results obtained by the proposed methods for the balanced dataset. Table 6 represents dataset in first column, obtained accuracy in second column and method results in the third. The hybrid method achieved 100% accuracy in both the training and testing phases, as represented by the blue bar. In contrast, the CNN method exhibited a few misclassifications, as indicated by the red bar in Fig. 4.

**Table 6:** Training, testing accuracy (in %).

Name of dataset	Accuracy	Name of the Method	
		LSTMCNN	CNN
Balanced dataset	Training Accuracy	100	99.47
	Testing Accuracy	100	99.3
Imbalanced Dataset	Training Accuracy	100	98.17
	Testing Accuracy	100	96.81

**Table 7:** Comparison of results for both datasets obtained using the LSTM-CNN hybrid method (in %).

Performance Metric	Balanced Dataset	Imbalanced Dataset
Accuracy	100	100
Precision	100	100
Recall	100	100
F Score	100	100

**Table 8:** Comparison of balanced dataset and imbalanced dataset for the CNN method (in %).

Performance Metric	Balanced Dataset	Imbalanced Dataset
Accuracy	99.47	98.17
Precision	99.49	98.23
Recall	99.48	98.17
F1 Score	99.48	98.17

Fig. 4 shows the results of the proposed methods for both datasets. In this case, the hybrid LSTM-CNN method performed better without biasing towards any class, achieving 100% accuracy. On the other hand, CNN method results were somewhat biased, with missclassification among the classes achieving 99.47% accuracy during the training of the balanced dataset and 98.17% accuracy for the imbalanced dataset..The CNN method obtained 99.30% and 96.81% accuracy during the testing of both datasets.

**Fig. 5:**
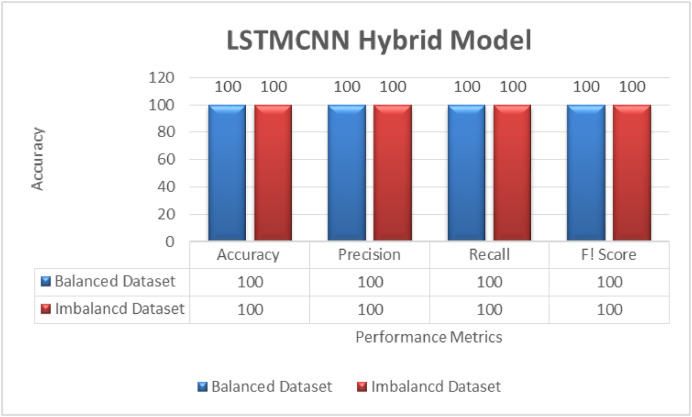
Summary of results obtained for LSTM-CNN hybrid method.

Fig. 5 and Table 7 represent the performance metrics of an LSTM-CNN hybrid method assessed on both balanced and imbalanced datasets. Table 7 represents performance metrics in the first column, followed obtained results for both datasets in the second and third columns. The proposed method achieves a perfect score of 1 across all metrics—accuracy, precision, recall, and F1 score—demonstrating outstanding performance. An accuracy of 1 indicates that the method correctly classifies every test sample. A precision of 1 means that all positive predictions are accurate, while a recall of 1 confirms that the method successfully identifies all actual positive cases. The F1 score, which is the harmonic mean of precision and recall, also reaches 1, indicating a perfect balance between these metrics. These results suggest that the method is exceptionally well-trained and highly generalizable. Fig. 5 shows the graphical representation of results for both datasets, the balanced dataset represented by the blue bar, and the imbalanced dataset represented by the red bar. Among these two methods implemented, the proposed LSTM-CNN method has achieved excellent results in terms of performance metrics.

**Fig. 6:**
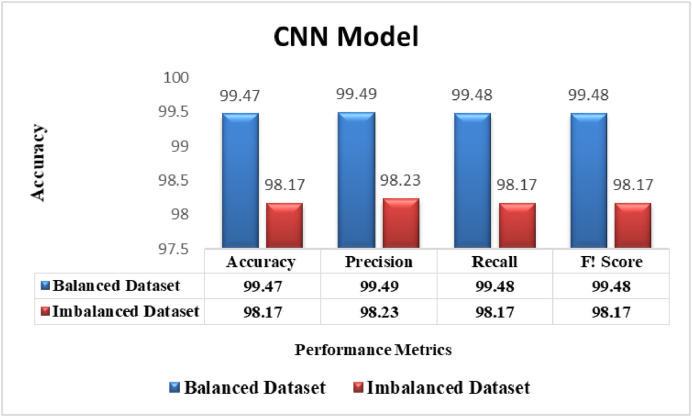
Summary of results obtained for the CNN method.

The CNN method performs well on both balanced and imbalanced datasets. There is a slight decrease in accuracy, precision, recall, and F1 score when working with an imbalanced dataset, as shown in Fig. 6 and Table 8. Table 7 represents performance metrics in the first column, followed obtained results for both datasets in the second and third columns for CNN method. This suggests that class imbalance affects the method's capacity to correctly detect instances of the minority class, which lowers overall performance. Compared to the LSTM-CNN hybrid method with an equivalent number of images in balanced and imbalanced datasets, CNN requires a comparatively large number of parameters during training.

The LSTM-CNN method surpasses the CNN method, achieving perfect scores (accuracy, precision, recall, and F1-score of 1) on balanced and imbalanced datasets. This demonstrates its exceptional ability to manage the imbalanced class. In contrast, the CNN method experiences a more significant decline in performance, with accuracy dropping from 99.47% to 98.17%, along with similar reductions in precision (from 99.49% to 98.23%), recall (from 99.48% to 98.17%), and F1-score (from 99.48% to 98.17%) for imbalanced dataset represented by red bar in Fig. 6 and Table 8. These findings indicate that LSTM-CNN is the most effective hybrid method for addressing imbalanced data.

**Fig. 7:**
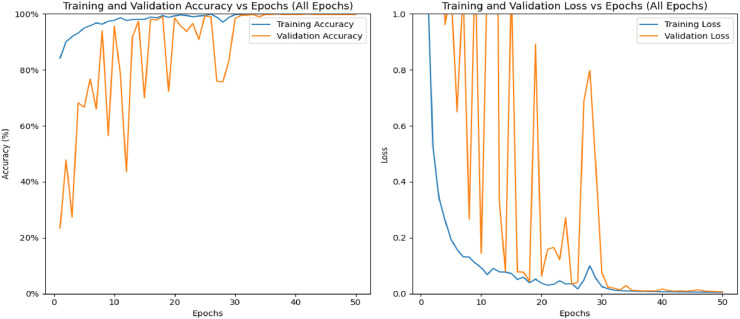
Accuracy and loss graph for balanced dataset – LSTM-CNN method.

**Fig. 8:**
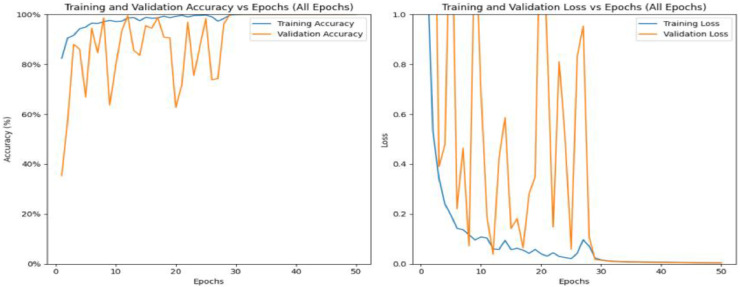
Accuracy and loss graph for imbalanced dataset – LSTM-CNN method.

Fig. 7, Fig. 8 show the accuracy and loss graph of the LSTM-CNN method. The left plot shows training and validation accuracy, where the training accuracy, represented by the blue line, steadily increases and reaches nearly 100%, indicating the model is learning well on the training data. However, the validation accuracy fluctuates significantly, represented by the red line. After 30 epochs, it becomes stable and achieves a 100% result. The right plot illustrates the training and validation loss trends, where training loss decreases smoothly, represented by the blue line, but validation loss, represented by the red line, exhibits sharp spikes and irregularity. The method fits the training data well, but validation loss has some fluctuations initially, but becomes stable after 30 epochs.

**Fig. 9:**
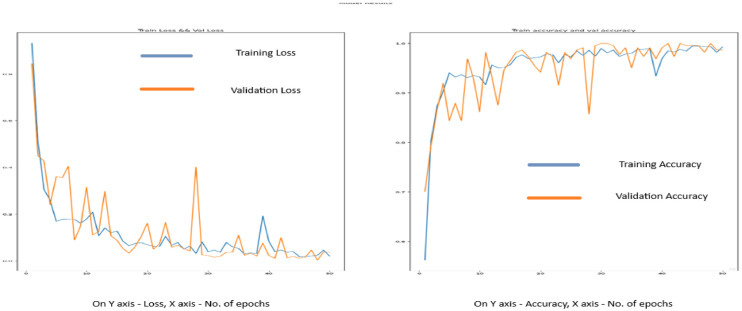
Accuracy and loss graph for balanced dataset – CNN method.

**Fig. 10:**
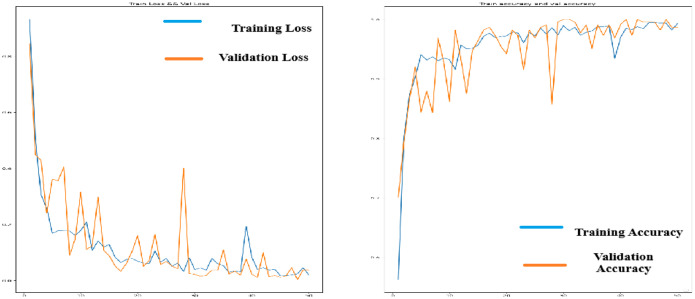
Accuracy and loss graph for imbalanced dataset – CNN method.

Figs. 9, 10 display the CNN method accuracy and loss graph, respectively. Less variation between training and validation parameters was seen during the implementation of this approach than with the LSTM-CNN method during training. Training and validation loss consistently drop in the left plot, which shows efficient learning and lowered prediction error across time. Although validation loss varies somewhat, generally it follows the training loss trend, indicating good generalization. The correct plot displays accuracy measures, in which case both training and validation accuracy fast rise and stabilize close to 100%. This indicates that the model learns effectively and performs precisely on both seen and unseen data. This CNN model shows more consistent and stable performance than the previous LSTM-CNN graph, so indicating it is well-optimized and less likely to overfit.

**Fig. 11:**
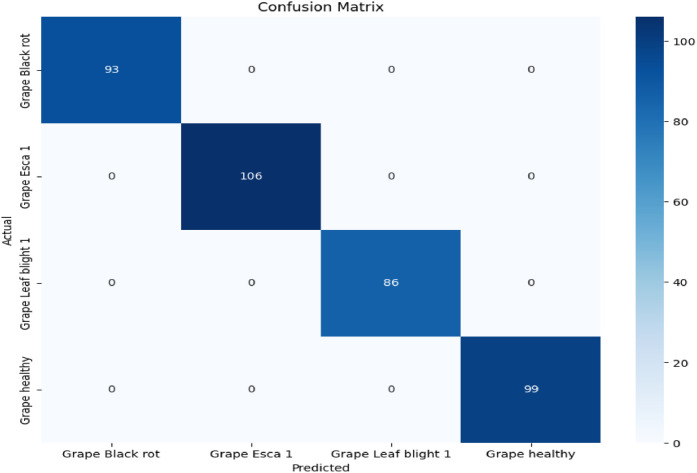
Confusion matrix LSTM-CNN method for balanced dataset.

**Fig. 12:**
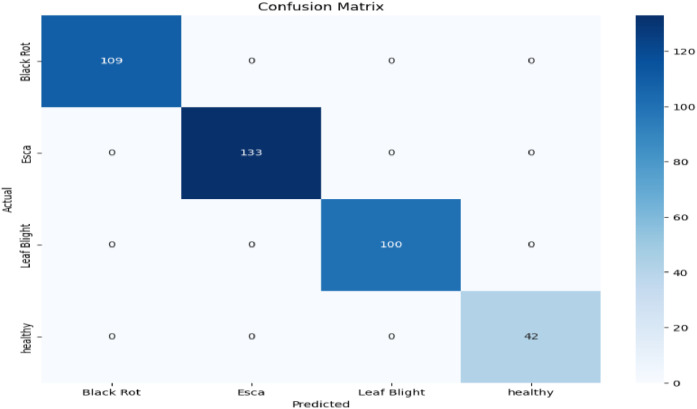
Confusion matrix LSTM-CNN method for imbalanced dataset.

Fig. 11, Fig. 12 show the confusion matrix for the LSTM-CNN hybrid method with both types of datasets. The Y-axis represents the actual class labels from the dataset, while the X-axis indicates the predicted outputs of the proposed methods. From the results, it has been observed that the proposed hybrid method outperforms in the prediction of disease classes. No image is misclassified, and no bias has been observed in both cases. In case of the balanced dataset, 93, 106, 86, and 99 images belong to Black Rot, ESCA, Leaf Blight, and Healthy classes, respectively, and are accurately classified by the proposed hybrid method. The imbalanced dataset with 109, 133, 100, and 42 belonging to Black Rot, ESCA, Leaf Blight, and Healthy classes is classified with 100% accuracy during the testing phase.

**Fig. 13:**
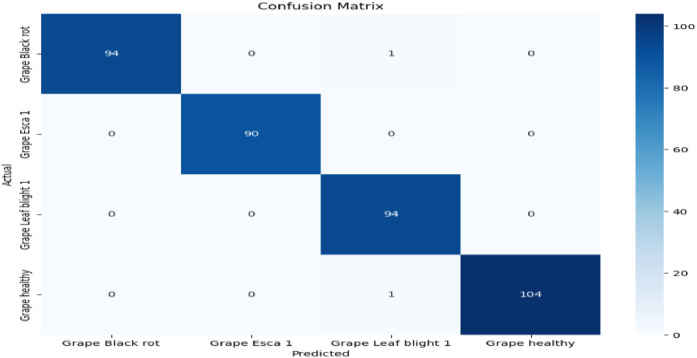
Confusion matrix for balanced dataset – CNN method.

**Fig. 14:**
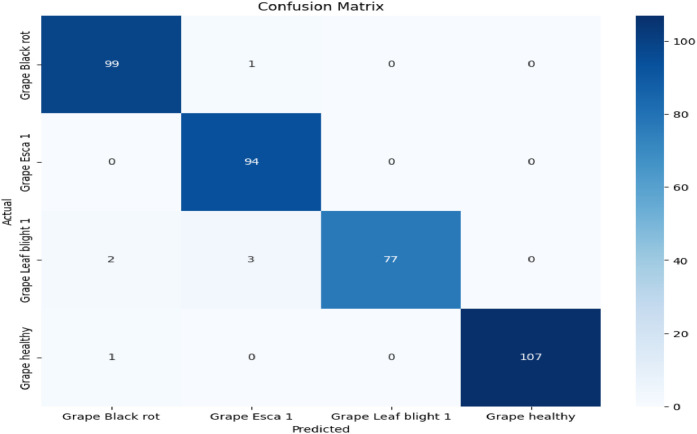
**C**onfusion matrix for imbalanced dataset – CNN method.

Fig. 13, Fig. 14 show the confusion matrix of the CNN method for grape crops. It shows that in both cases, a slight misclassification is observed. In the balanced dataset, 1 image belonging to the leaf blight class is classified as grape healthy, and 1 image from the Black rot class is classified as leaf blight. On the other hand, 3 images from leaf blight are classified as Esca class when the imbalanced dataset is tested. One image from healthy and black rot is classified as black rot and esca, respectively. Otherwise, the methods perform well in both cases.

When these two proposed methods are compared, the LSTM-CNN hybrid methods outperform the CNN method in both datasets, i.e., Balanced and Imbalanced.

### Comparison with SOTA methods

**Fig. 15:**
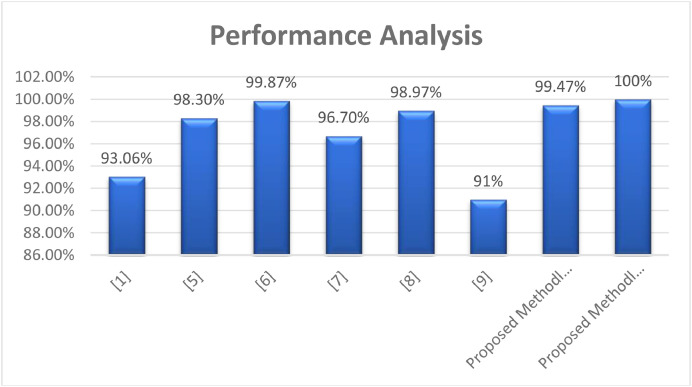
Comparison with SOTA methods.

The proposed methods are compared with the SOTA methods implemented for the grape leaf classification and detection. Table 9 and Fig. 15 show that our proposed hybrid method outperforms the existing SOTA methods for grape disease classification.

**Table 9:** Comparison of proposed models with existing methods.

Reference	Method Used	Name of the Crop	Accuracy
[1]	CNN LSTM Hybrid Method	Grape	93.06%
[5]	MobileNetV2 encoder	Rice, Wheat, Tea, Banana, and Sugarcane	98.3%
[6]	Enhanced CNN	Multiple Crops	99.87%
[7]	ResNET-50	Grapes	96.7%
[8]	Multiclass SVM	Grapes	98.97%
[9]	Multimodal data and parallel heterogeneous activation functions	Grapes	91%
Proposed Model - II	Standalone CNN Method	Grapes	99.47%
Proposed Model - I	LSTM and CNN-based Hybrid Method	Grapes	100%

### Statistical analysis

**Fig. 16:**
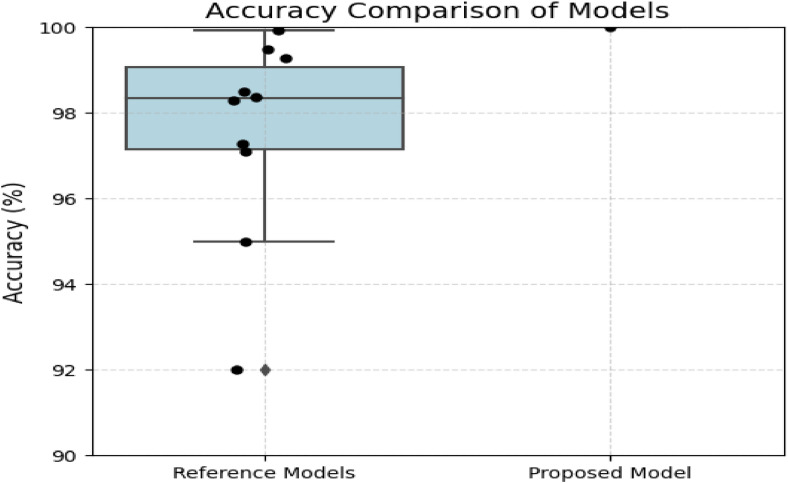
Statistical analysis of proposed work using the Wilcoxon Signed-Rank test.

A Wilcoxon Signed-Rank test on paired results from 7 samples before and after using the suggested technique shown in Fig. 16 helps to statistically validate the performance improvement of the proposed grape disease classification and detection method. The small sample size and possible non-normal distribution of the performance measures led to the choice of the non-parametric test. With a p-value of 0.016 and a statistic of W = 0.0, the test indicates a statistically significant difference at the 5% significance level. This outcome shows consistent and appreciable improvements over all tested samples since it confirms that the suggested approach considerably raised the classification accuracy over the baseline.

## Conclusion

Two separate deep learning architectures—a hybrid Long Short-Term Memory–Convolutional Neural Network (LSTM-CNN) method and a conventional Convolutional Neural Network (CNN) method—are compared in this work. For both used datasets—balanced and imbalanced—the hybrid approach has attained an overall accuracy of 100%. Key variations in training stability and generalization between the LSTM-CNN and CNN models are found by means of a comparative analysis. Although the LSTM-CNN method achieves 100% accuracy, its validation accuracy and loss graphs show notable fluctuations in the first epochs, indicating somewhat instability during training, and it stabilizes after 30 epochs, so obtaining a perfect score. Additionally, the computing cost has been reduced by 476,703 parameters for the LSTM-CNN method, compared to the CNN method, which has 3546,896 parameters. The hybrid method has reduced the computational cost by 3.7 M parameters. By contrast, the CNN method shows smoother and more consistent trends in both accuracy and loss curves for training and validation even if it achieves somewhat lower peak accuracy at 99.47%. This implies that one can implement the LSTM-CNN and CNN approaches in real-time. With less variance over epochs, both techniques are more robust and generalize better.

### Limitations

Although the LSTM-CNN method has shown impressive results, several potential limitations of current methods could be considered for future work that could further improve its performance and usability:

• Generalization to Other Crops

Currently, the method is tailored to detect diseases in grape crops. It could be adapted for multi-crops (such as apples, potatoes, tomatoes, and sugarcane) to create a more universal plant disease detection system.

## Ethics statements

This article does not contain any studies with human or animal participants.

## Funding

This research received no external funding.

## CRediT author statement

**Vinod Mulik**: Conceptualization, Methodology, Software, Data curation, Investigation, Writing, Original draft preparation. **Dr. Vinod Patil**: Supervision, Writing, Reviewing, and Editing.

## Related research article

**None.**

## Declaration of competing interest

The authors declare that they have no known competing financial interests or personal relationships that could have appeared to influence the work reported in this paper.

## Data Availability

Data will be made available on request.

## Appendix Supplementary materials

Image, application 1

## References

[1] Gaur P., Sharma R., Kumar R., Gupta A., Sharma R., Kukreja V. (2023). 2023 4th International Conference for Emerging Technology (INCET).

[2] Sharma A., Kumar V., Shahzad B. (2019). Worldwide pesticide usage and its impacts on the ecosystem. SN Appl. Sci..

[3] Kunduracioglu I., Pacal I. (2024). Advancements in deep learning for accurate classification of grape leaves and diagnosis of grape diseases. J. Plant Dis. Prot..

[4] Chen Z., Feng J., Zhu K., Yang Z., Wang Y., Ren M. (2024). YOLOv8-ACCW: lightweight grape leaf disease detection method based on improved YOLOv8. IEEE Access.

[5] Karthik R. (2024). GrapeLeafNet: a dual-track feature fusion network with inception-ResNet and shuffle-transformer for accurate grape leaf disease identification. IEEE Access.

[6] Thanjaivadivel M., Gobinath C., Vellingiri J. (2025). EnConv: enhanced CNN for leaf disease classification. J. Plant Dis. Prot..

[7] Zhou C., Zhang Z., Zhou S., Xing J., Wu Q., Song J. (2021). Grape leaf spot identification under limited samples by fine grained-GAN. IEEE Access.

[8] Javidan Seyed Mohamad, Banakar Ahmad, Vakilian Keyvan Asefpour, Ampatzidis Yiannis (2023). Diagnosis of grape leaf diseases using automatic K-means clustering and machine learning. Smart Agric. Technol..

[9] Li R., Liu J., Shi B., Zhao H., Li Y., Zheng X., Peng C., Lv C. (2024). High-performance grape disease detection method using multimodal data and parallel activation functions. Plants.

[10] Padol P.B., Yadav A.A. (2016). 2016 Conference on Advances in Signal Processing (CASP).

