# Supplementary material for: Enhancing grape disease detection: A comparative analysis of hybrid CNN-LSTM and CNN methods
Source: MethodsX. 2026 Jun 2;16:103983. doi: 10.1016/j.mex.2026.103983 (PMC13251194; doi:10.1016/j.mex.2026.103983)
Supplement: Supplementary file 1 [file mmc1.docx]

Details of the Dataset –

1. **Summary of the dataset Used**

Summary of Grape Dataset

| Dataset Type | No. of Images | Healthy Class | ESCA Class | Leaf Blight Class | Black Rot Class |
| --- | --- | --- | --- | --- | --- |
| Balanced Dataset | 4000 | 1000 | 1000 | 1000 | 1000 |
| Imbalanced Dataset | 4062 | 423 | 1383 | 1076 | 969 |

1. **The details of the disease on grape leaves.**

| Name of the Grape Leaf Disease Class | Cause | Symptoms | Timing |
| --- | --- | --- | --- |
| ESCA | A complex disease involving multiple fungal species | Interveinal "striping": Dark red stripes on red grape varieties and yellow stripes on white varieties | More common during July and August, but can affect leaves at any time |
| Leaf Blight | Often caused by fungi like Exserohilum turcicum | Small yellow dots along leaf margins that expand into brown patches.  Can appear as yellowish spots with concentric rings | Often occurs in high humidity conditions |
| Black Rot | Primarily caused by the fungus Guignardia bidwellii. | On leaves: Irregularly shaped reddish-brown spots that may coalesce into larger blotches | New growth on the vine is most susceptible during the growing season, but berries can be infected until almost fully grown, especially without fungicide protection |
| Healthy | NA | No visible signs of disease or stress represent the plant's standard physiological condition. | NA |

1. **Architecture or tentative block diagram of the proposed method for implementation and its flow** **of implementation**
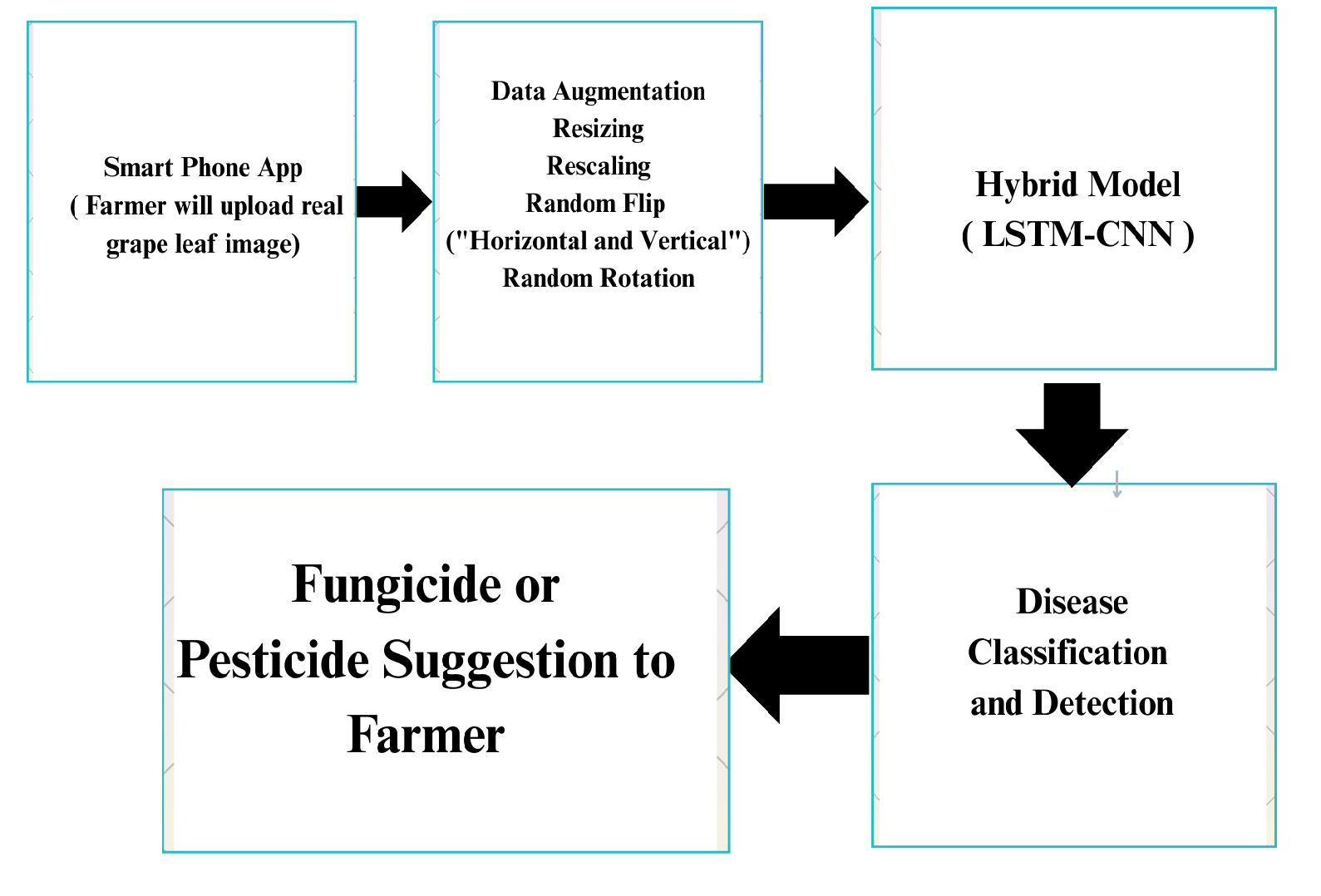


This diagram outlines a **practical AI-driven pipeline** for **grape leaf disease diagnosis** and **treatment recommendation**, integrating mobile technology, image processing, and machine learning (LSTM-CNN hybrid model). Here's a **detailed explanation from an agronomist's point of view**, along with **practical guidance** on how this can be implemented in real-world scenarios:

**1. Smartphone App (Input Stage)**

*“Farmer/Agronomist will upload a real grape leaf image.”*

**Explanation:**
Farmers use a smartphone application to **capture and upload images of grape leaves** from their vineyards. The goal is to identify visible symptoms of diseases (e.g., black rot, , downy mildew).

**Practical Guidance:**

- Develop a **user-friendly mobile app** (Android/iOS) in local languages with minimal data usage.
- Ensure the app allows farmers to:
  - Capture high-resolution images.
  - Submit metadata (e.g., location, variety, age of crop).
- Add **simple image guidelines** (e.g., take close-up, clear photos in good lighting).
- Include offline capability so farmers can upload when internet is available.

**2. Data Pre-processing (Image Augmentation)**

**Label:** *Data Augmentation, Resizing, Rescaling, Random Flip, Random Rotation*

**Explanation:**
Uploaded images undergo pre-processing to **enhance the dataset** by simulating different scenarios (like angle, lighting). This improves the robustness of the model.

**Processes Include:**

- **Resizing**: All images resized to a uniform shape (e.g., 224x224).
- **Rescaling**: Pixel values normalized (e.g., 0–1 scale).
- **Flipping/Rotating**: Images randomly flipped or rotated to mimic various viewing angles.

**Practical Guidance:**

- Use libraries like TensorFlow/Keras or PyTorch to automate this augmentation.
- Maintain a **balanced dataset** with equal representation of all disease types to avoid bias.
- Store augmented images securely and ensure privacy compliance for farmers.

**3. Hybrid Model (LSTM-CNN)**

**Label:** *Hybrid Model (LSTM-CNN)*

**Explanation:**
This hybrid model combines:

- **CNN (Convolutional Neural Network)**: Extracts spatial features (spots, color changes, edge patterns) from leaf images.
- **LSTM (Long Short-Term Memory)**: Captures **temporal dependencies or sequential patterns**, useful if multiple images over time are provided.

**Why Hybrid?**

- CNN handles image patterns effectively.
- LSTM helps track **disease progression over time** if farmers upload periodic images.

**Practical Guidance:**

- Train the model using the **PlantVillage dataset** or your own **annotated grape disease dataset**.
- Fine-tune the model using real field data (farmers' contributions).
- Evaluate model accuracy using metrics like precision, recall, and F1-score.

**4. Disease Classification and Detection**

**Label:** *Disease Classification and Detection*

**Explanation:**
The model classifies the leaf as:

- Healthy
- Infected with a specific disease (e.g., downy mildew, black rot, etc.)

**Practical Guidance:**

- Set thresholds for confidence scores (e.g., ≥90% confidence for reliable prediction).
- Provide visual feedback to farmers (e.g., “Your grape leaf is infected with downy mildew”).

**5. Fungicide or Pesticide Suggestion**

**Label:** *Fungicide or Pesticide Suggestion to Farmer*

**Explanation:**
Based on the disease identified, the system recommends:

- **Name of fungicide or pesticide**
- **Dosage and spray frequency**
- **Precautionary measures**

**Practical Guidance:**

- Collaborate with **agricultural universities or local agronomists** to compile a list of **region-specific, government-approved** pesticide recommendations.
- Recommendations should include:
  - Brand name & generic name
  - Dosage per liter
  - Best time of application
  - Organic alternatives (if available)
- Optionally, integrate with **e-commerce platforms or local agro-dealers** to show availability.

**Summary: End-to-End Practical Flow**

| **Stage** | **Task** |
| --- | --- |
| **1. Image Input** | Farmer captures grape leaf via mobile app |
| **2. Pre-processing** | Image resizing, flipping, rotating (via app or backend server) |
| **3. Hybrid Model** | LSTM-CNN classifies disease from uploaded image |
| **4. Detection Output** | Output shows name and severity of the disease |
| **5. Recommendation** | Suggest specific treatment (chemical/organic), dosage, and schedule |

**Tools & Technologies You Can Use:**

- **Mobile App:** Flutter or React Native
- **Backend Server:** Flask / Django for image processing
- **ML Model:** TensorFlow/Keras (for LSTM-CNN)
- **Deployment:** Firebase, AWS, or Azure
- **Database:** Firebase Firestore or PostgreSQL for farmer data

**Agronomist's Role in Implementation:**

- **Data Annotation**: Help tag disease images for supervised learning.
- **Validation**: Approve model predictions against ground truth.
- **Training & Outreach**: Train farmers to use the app and interpret results.
- **Feedback Loop**: Collect disease progression data to retrain and improve model.
